# Stress granules inhibit apoptosis by reducing ROS production, but this phenomenon is nullified by HTLV-1 Tax

**DOI:** 10.1186/1742-4690-11-S1-O52

**Published:** 2014-01-07

**Authors:** Masahiko Takahashi, Masaya Higuchi, Masahiro Fujii

**Affiliations:** 1 Division of Virology, Niigata University Graduate School of Medical and Dental Sciences, Niigata, Japan

## 

Human T-cell leukemia virus type 1 (HTLV-1) is the etiological agent of adult T-cell leukemia (ATL), and the viral oncoprotein Tax plays key roles in immortalization of human T-cells and leukemogenesis. Here, we identified the ubiquitin-specific protease 10 (USP10) as a Tax-interactor in T-cells. Upon an exposure to arsenic, an oxidative stress inducer, USP10 was recruited into stress granules (SGs), which are the anti-stress machinery. USP10-knockout indicated that USP10, through augmenting formation of SGs, reduced reactive oxygen species (ROS) production and inhibited ROS-dependent apoptosis. Tax mutants and USP10-knockdown indicated that Tax inhibits arsenic-induced SG formation, stimulates ROS production and augments ROS-dependent apoptosis, and they are all dependent on interactions of Tax with USP10. These findings suggest that USP10 is a host factor that controls stress-induced ROS production and apoptosis in HTLV-1-infected T-cells. A clinical trial showed that a combination therapy containing arsenic is effective against some forms of ATL. Thus, these findings may also be relevant to chemotherapy against ATL.

